# NAMPT Impairs Vascular Permeability in Periodontitis by Influencing FASN-mediated Lipogenesis

**DOI:** 10.7150/ijbs.104485

**Published:** 2025-03-31

**Authors:** Yijing Xiao, Jiahui Ma, Zirui Li, Huiqing Gou, Xu Chen, Yi Zhou, Ming Shen, Lu Li, Yan Xu

**Affiliations:** 1 Department of Periodontology, The Affiliated Stomatological Hospital of Nanjing Medical University, Nanjing, China; 2 State Key Laboratory Cultivation Base of Research, Prevention and Treatment for Oral Diseases (Nanjing Medical University), Nanjing, China; 3 Jiangsu Province Engineering Research Center of Stomatological Translational Medicine, Nanjing, China.; 4 Department of General Dentistry, The Affiliated Stomatological Hospital of Nanjing Medical University, Nanjing, China.

**Keywords:** NAMPT, FASN, vascular permeability, transendothelial migration, periodontitis

## Abstract

Vascular abnormalities promote tissue inflammation and bone loss. Although vascular abnormalities in periodontitis have been studied, underlying pathogenic mechanisms remain unclear. This study aimed to investigate key molecules regulating endothelial cell permeability and explore their role in the progression of periodontitis. Single-cell RNA sequencing revealed leukocyte transendothelial migration in periodontitis is associated with endothelial cells. Moreover, increased vascular permeability was observed in both human and mouse periodontitis tissues. Nicotinamide phosphoribosyltransferase (NAMPT) protein expression was significantly upregulated in endothelial cells within periodontitis tissues, with levels increasing as the disease progressed. NAMPT gain-of-function decreased VE-cadherin expression and membrane potential, increased HUVEC permeability, and promoted leukocyte trans-endothelial migration. Mechanically, NAMPT elevated levels of triglycerides and free fatty acids, leading to lipid droplet accumulation in HUVEC. Fatty acid synthase (FASN), an enzyme that catalyzes the biosynthesis of fatty acids, is also raised with NAMPT. NAMPT promoted NADPH pool which is utilized in FASN-mediated lipogenesis. FASN inhibitor orlistat reversed lipogenesis and endothelial permeability induced by NAMPT. Furthermore, orlistat administration reduced periodontal vascular permeability and further reversed bone resorption in periodontitis mice. This study demonstrated that increased NAMPT in periodontitis promotes endothelial permeability by modulating FASN-mediated lipogenesis, thereby contributing to bone loss in periodontitis.

## Introduction

Periodontitis is a chronic inflammatory disease that occurs in the periodontal supporting tissues and is characterized by the destruction of the periodontal ligament and pathological loss of alveolar bone^1^. Similar to other inflammatory disorders, the infiltration and hyperactivation of immune cells promote the progression of periodontitis^2^, ^3^. Blood vessels serve as conduits for immune cells, facilitating their migration to sites of inflammation through a process known as trans-endothelial migration (TEM)^4^. Increased endothelial cell permeability enhances leukocyte TEM and promotes tissue inflammation^5^. It has been reported that pathogenic factors of periodontitis enhance vascular permeability^6^. One of the most significant pathogenic factors, Porphyromonas gingivalis' outer membrane vesicles (P.g-OMVs), increase permeability of microvascular endothelial cells (HMEC-1) by decreasing intercellular adhesion molecule PECAM-1 (CD31)^7^. Additionally, Porphyromonas gingivalis (P.g) has been shown to increase HUVEC permeability and further regulate leukocyte trans-endothelial migration (TEM) in vitro^8^. However, the effects of periodontitis on blood vessels are often studied in systemic diseases such as retinal disease and cardiovascular disease, to confirm the regulatory role of periodontitis on the whole body^7^, ^9^, the impact of local vascular changes in periodontal tissue on the progression of bone resorption in periodontitis and its pathogenic mechanism has rarely been studied.

As a key enzyme regulating intracellular nicotinamide adenine dinucleotide (NAD+) synthesis, Nicotinamide phosphoribosyltransferase (NAMPT) mainly regulates the activity of NAD-dependent enzymes such as sirtuins to suppress cell senescence^10^, ^11^. Beyond its role in cellular NAD+ metabolism, emerging evidence suggests that NAMPT also plays a significant role in inflammation^12^, ^13^. NAMPT inhibition could alleviate intestinal inflammation by reducing macrophage infiltration and polarization^14^. Furthermore, NAMPT is overexpressed in both rheumatoid arthritis (RA)-mediated periodontitis and inflamed human and mouse gingival tissues^15^, ^16^. However, the role of NAMPT in periodontal endothelial cells remains unexplored.

Abnormal lipid metabolism is associated with many diseases, including cardiovascular dysfunction, chronic kidney disease, fatty liver, obesity, neurodegeneration, and cancer^17^. An increase in lipid droplet formation has been observed in LPS-induced endothelial inflammation^18^. Additionally, palmitic acid-induced lipid accumulation leads to endothelial dysfunction by elevating inflammation and oxidative stress^19^. Fatty acid synthase (FASN) catalyzes a series of reactions that convert acetyl-CoA and malonyl-CoA into fatty acids, which is important in lipid synthesis. It has been reported that FASN is involved in endothelial dysfunction. Decreasing the level of FASN ameliorates retinal vascular dysfunction in diabetes mellitus^20^. FASN inhibition alleviated the exacerbation of LPS-induced lung injury under obesity via rescuing lung vascular leakage^21^.

In this study, we aim to investigate the increased vascular permeability associated with periodontal disease, with a particular focus on abnormal lipid metabolism due to NAMPT. By elucidating the regulatory mechanism of NAMPT on FASN and exploring the inhibitory effect of FASN inhibitors on vascular permeability and bone resorption in periodontitis, we aim to gain new insights into periodontitis and further shed light on potential therapeutic strategies for managing periodontal disease.

## Results

### Vascular permeability increased to promote TEM in periodontitis

**Figure 1:**
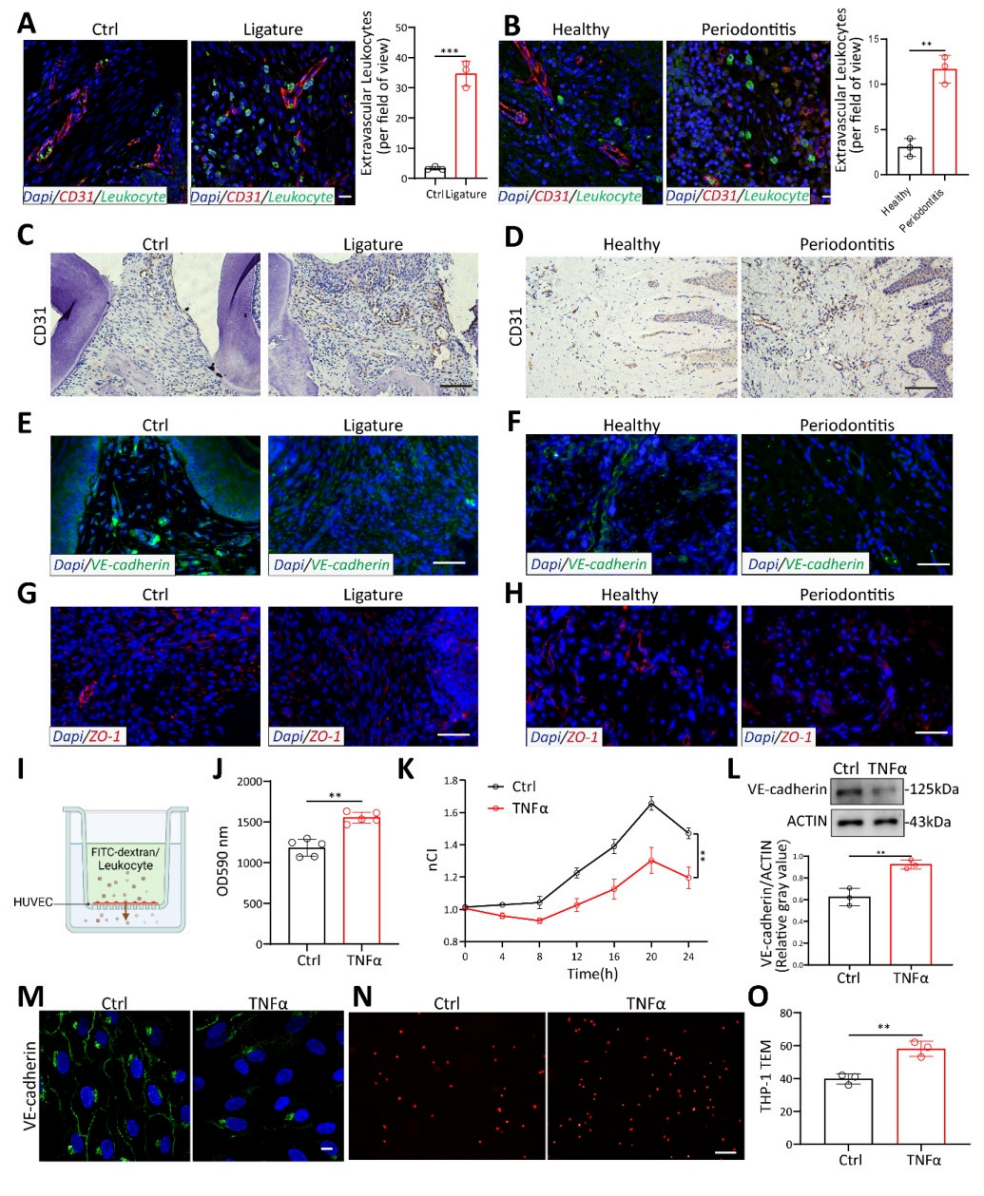
** Vascular permeability increased to promote TEM in periodontitis.** (A-B) Laser confocal microscope observation of S100A9(green) labeled neutrophils transmitted through CD31(red) labeled vessels in periodontitis mice and patients. Scale bars: 20µm, n=3. (C-D) Immunohistochemistry of CD31 in periodontitis mice and patients. Scale bars: 100µm. (E-F) Immunofluorescence of the junction marker VE-cadherin(green) in periodontitis mice and patients. Scale bars: 100µm. (G-H) Immunofluorescence of the junction marker ZO-1(red) in periodontitis mice and patients. Scale bars: 100µm. (I) In vitro permeability and TEM assay model. (J) FITC-dextran density permeated across HUVEC after exposure to TNFα measured by Spectra Max microplate reader, n=5. (K) The impedance of HUVECs in the RTCA system after TNFα treatment at different time points, n=3. (L) Western blot analysis and quantification of VE-cadherin in TNFα-treated HUVECs, n=3. (M) Immunofluorescence of VE-cadherin in TNFα-treated HUVECs. Scale bars: 10µm. (N-O) Representative images and quantitative analysis of Dil-labeled THP-1 cells transmigrating through endothelial cells after TNFα stimulation, n=3. Scale bars: 100µm. Error bars indicate SEM. Two-tailed unpaired Student's *t* test was performed. **P < 0.01, ***P < 0.001.

To investigate the pathways associated with periodontitis, we analyzed single-cell RNA sequencing (scRNA-seq) dataset GSE164241, which includes gingival tissue samples from 8 periodontitis patients and 13 healthy donors. All data were divided into 9 sub-clusters (Figure S1A). All marker genes were displayed in a dot plot (Figure S1B). Top Kyoto Encyclopedia of Genes and Genomes (KEGG) pathway enrichment analysis revealed significant upregulation in pathways related to pathogenic infection, oxidative phosphorylation, leukocyte transendothelial migration (TEM), and protein processing in the endoplasmic reticulum when comparing periodontitis samples to healthy controls (Figure S1C). These findings align with the known pathological mechanisms of periodontitis. Leukocytes are key cells involved in periodontitis, responsible for destroying bone tissue and phagocytizing bacteria along with other inflammatory agents^3^. Leukocyte TEM, crucial for immune cell infiltration into inflamed tissues, has rarely been studied in periodontitis. To investigate the role of leukocyte TEM in periodontitis, experimental periodontitis was induced in C57BL/6 mice by bilateral maxillary molar ligation for 14 days, and clinical samples were collected. Alveolar bone loss was significantly greater in ligature-induced periodontitis mice compared to control mice (Figure S2A-D). Inflammatory cytokines, including TNFα and IL-6, as well as F4/80-labeled macrophages, were significantly elevated in both human and mouse periodontal tissues, confirming the presence of a robust inflammatory environment (Figure S2E-J). Additionally, increased infiltration of S100A9-labeled leukocytes was observed in the periodontitis mice (Figure 1A). Similarly, clinical samples from periodontitis patients exhibited a significant rise in leukocyte TEM (Figure 1B).

Given the involvement of both the opening of the endothelial barrier and immune cell migration^4^, we aim to identify which cell types are affected and contribute to TEM in periodontitis. KEGG pathway enrichment analysis of endothelial and monocyte/macrophage subclusters in scRNA sequencing revealed that the TEM pathway was predominantly enriched in endothelial subcluster, rather than in monocytes/macrophages (Figure S1D-E). This suggests that the enhanced TEM pathway in periodontitis primarily affects endothelial cells, while its impact on macrophages is more related to metabolic changes and polarization, which aligns with previous studies^22^, ^23^. These findings indicate that vascular endothelial cells may play a key role in facilitating the leukocytes TEM in periodontitis. To investigate vascular changes in periodontitis, IHC staining of blood vessels labeled with CD31 was performed. The results revealed marked proliferation and dilation of vessels in both periodontitis patients and mice (Figure 1C-D). Additionally, fluorescence intensity of VE-cadherin, an endothelium-specific adhesion molecule, was decreased in gingival tissues from both mice and patients with periodontitis (Figure 1E-F). The tight junction protein ZO-1 was also reduced in periodontitis (Figure 1G-H). To further evaluate vascular permeability in periodontitis, Evans Blue dye was administered via tail vein injection in mice. In periodontitis mice, vascular permeability was significantly elevated, as indicated by the increased penetration of Evans Blue (EB) dye into the periodontal tissue (Figure S2K-L). These findings highlight impaired vascular permeability in periodontitis.

To further confirm the role of inflammation on endothelial cells (ECs), we stimulated HUVECs with TNFα, the most commonly used cytokine for inducing endothelial inflammation. FITC-dextran was added to the upper well and allowed to transmit to the lower chamber through HUVECs (Figure 1I). Fluorescence intensity increased significantly with TNFα stimulation, indicating that more FITC-dextran permeates across the endothelium (Figure 1J). To better investigate the effect of TNFα on HUVEC permeability, real-time cell analysis (RTCA) was performed. Changes in cell membrane potential were detected every 15 minutes after plateau (Figure S2M). TNFα decreased the membrane potential of HUVEC over time (Figure 1K). Western blot and immunofluorescence showed that VE-cadherin decreased at HUVEC cell junctions under TNFα stimulation (Figure 1L-M). Disruption of endothelial cell junctions has been reported to lead to excessive leukocyte TEM and increased tissue destruction^5^. To investigate the impact of endothelial cell permeability on leukocyte TEM, Dil-labeled THP-1 monocytes were added to a confluent monolayer of endothelial cells and co-cultured for 4 hours (Figure 1I). The results revealed that TNFα stimulation significantly increased the number of monocytes transmigrating through the HUVEC monolayer (Figure 1N-O). This demonstrates that an inflammatory environment in periodontitis can promote endothelial cell permeability, thereby increasing leukocyte TEM.

### NAMPT is highly expressed in the endothelial cells of periodontitis, facilitating increased endothelial cell permeability

**Figure 2:**
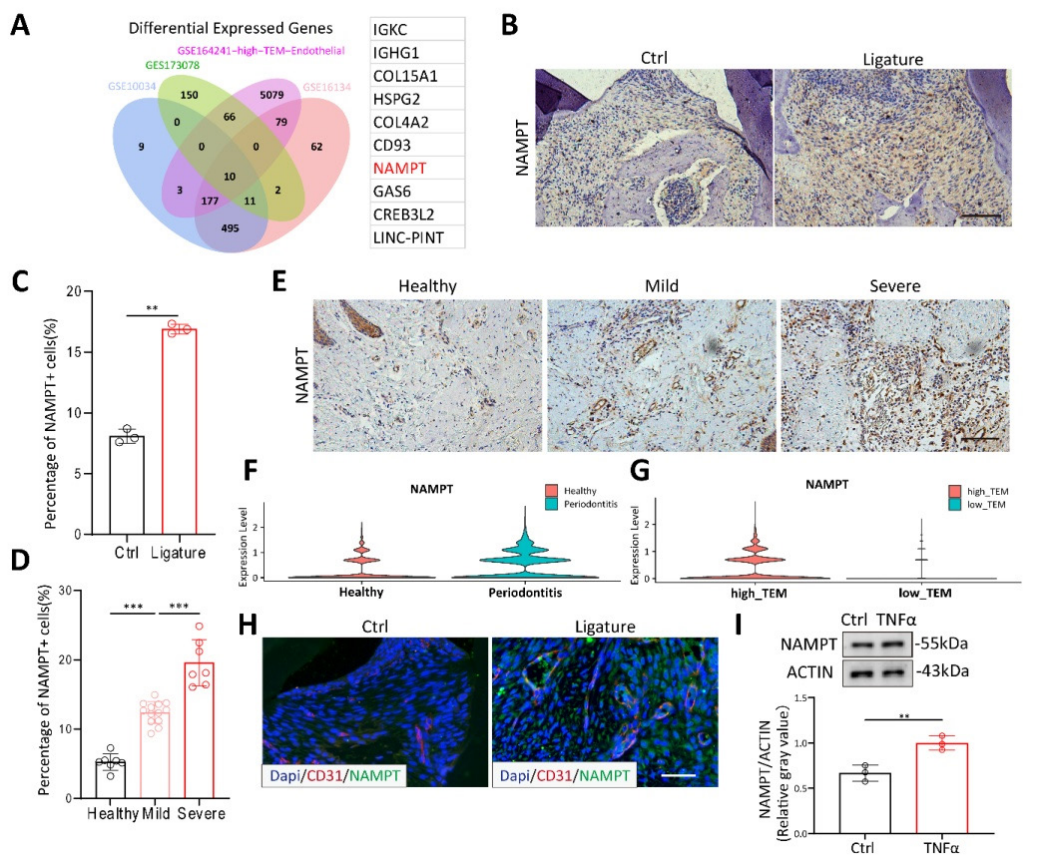
**NAMPT is highly expressed in the endothelial cells of periodontitis.** (A) Venn diagram showing the overlap of highly expressed genes across the four databases, accompanied by the gene list on the right. (B-C) Immunohistochemistry staining of NAMPT in periodontitis mice with quantifications, n=3. Scale bars: 100µm. (D-E) Immunohistochemistry staining of NAMPT in periodontitis patients(n=20) at different stage with quantifications. Scale bars: 100µm. (F)Violin plot displaying the distribution of NAMPT expression across endothelial sub-clusters in both healthy and periodontitis samples. (G) Violin plot comparing NAMPT expression levels in endothelial sub-clusters between high-TEM and low-TEM. (H) Immunofluorescence of NAMPT (green) and CD31(red) in periodontitis mice. Scale bars: 100µm. (I) Western blot analysis and quantification of NAMPT in TNFα treated HUVECs, n=3. Error bars indicate SEM. Two-tailed unpaired Student's *t* test was performed between two groups. For multiple comparisons, one-way ANOVA followed by Turkey's test was used. **P < 0.01, ***P < 0.001.

To explore the key molecules affecting periodontitis, we obtained three datasets GSE173078, GSE16134 and GSE10334 from GEO database, comprising 436 periodontitis samples and 145 healthy controls. Endothelial subcluster of GSE164241 was re-clustered with TEM pathway. By intersecting these datasets, we identified 10 significantly upregulated genes (Figure 2A). NAMPT, a molecule known to be associated with various inflammatory diseases in recent years^14^,^24^, has garnered our attention. PCR results showed that NAMPT changed most significantly under TNFα stimulation (data not shown). Thus, to further confirm the effect of NAMPT, we examined its expression in mice and patients with periodontitis using IHC staining. NAMPT expression was significantly elevated in periodontitis mice compared to healthy controls (Figure 2B-C). Moreover, in clinical samples from patients at different stages of periodontitis, NAMPT levels showed a progressive increase in accordance with disease severity (Figure 2D-E). These findings suggest that NAMPT could be an important marker for periodontitis. Additionally, NAMPT was significantly upregulated in the endothelial cell subpopulation of periodontitis and positively correlated with TEM (Figure 2F-G). Immunofluorescence staining further confirmed NAMPT colocalization with CD31+ endothelial cells (Figure 2H). Moreover, TNFα stimulation was shown to increase NAMPT expression in HUVECs (Figure 2I). Overall, these results suggest that NAMPT is highly expressed in endothelial cells during periodontitis and may play a key role in modulating the TEM process in the disease.

**Figure 3:**
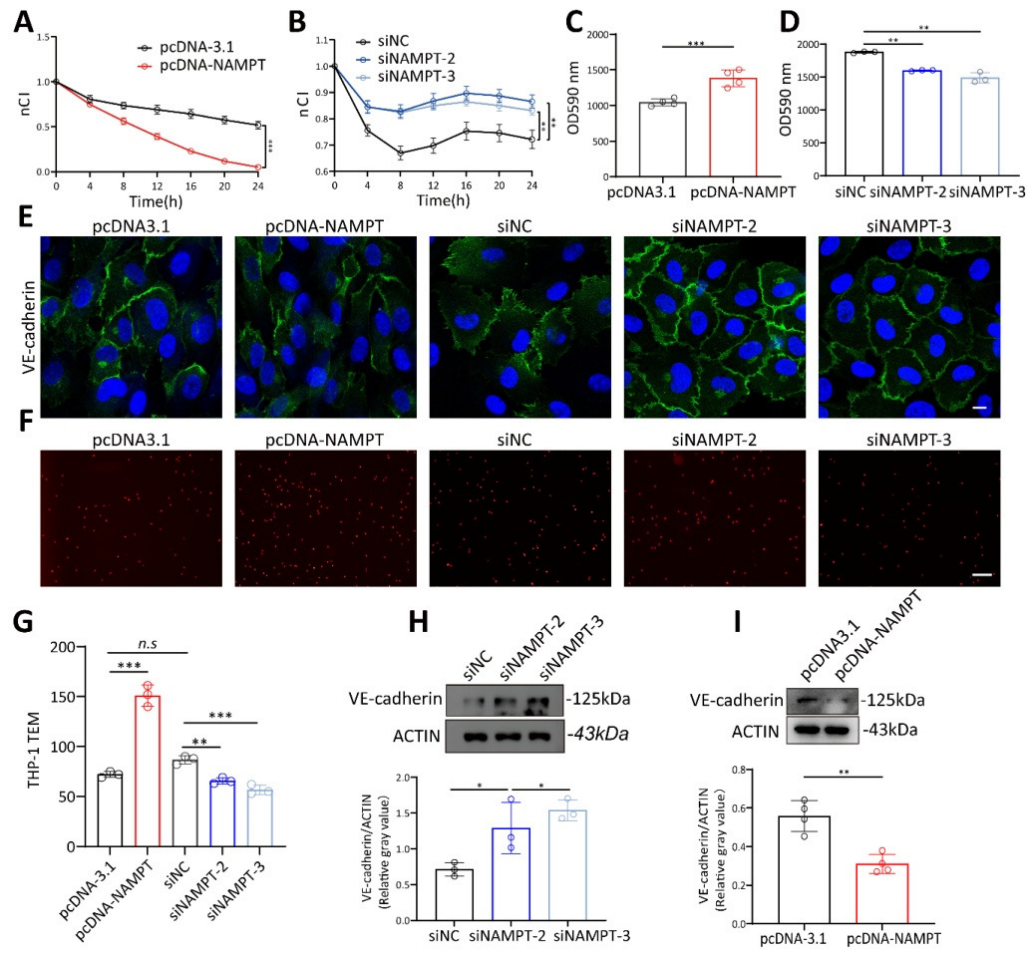
**NAMPT increased HUVEC permeability.** (A) The effect of NAMPT overexpression on HUVEC impedance was assessed using the RTCA system, with measurements taken at multiple time points, n=3. (B) The effect of NAMPT knockdown on HUVEC impedance was assessed using the RTCA system, with measurements taken at multiple time points, n=3. (C) FITC-dextran density permeated across HUVEC monolayers after NAMPT overexpression, n=4. (D) FITC-dextran density permeated across HUVEC monolayers after NAMPT knockdown, n=3. (E) Immunofluorescence analysis of VE-cadherin in NAMPT-overexpressing and knockdown HUVECs. Scale bars: 10µm. (F-G) Representative images and quantitative analysis of Dil-labeled THP-1 cells passing through endothelial cells after NAMPT overexpression and knockdown, n=3. Scale bars: 20µm. (H-I) Western blot analysis and quantification of VE-cadherin in NAMPT-overexpressing (n=4) and knockdown (n=3) HUVECs. Error bars indicate SEM. Two-tailed unpaired Student's *t* test was performed between two groups. For multiple comparisons, one-way ANOVA followed by Turkey's test was used. n.s, not significant, *P < 0.05, **P < 0.01, ***P < 0.001.

To verify the role of NAMPT in regulating endothelial cell permeability, NAMPT plasmid and siRNA were constructed and transfected into HUVECs. Transfection efficiency was detected by RT-qPCR and WB (Figure S3A-D). siNAMPT-2 and siNAMPT-3 were further used for the following experiments based on the knockout efficiency. Results showed that higher NAMPT expression led to a decrease in HUVEC membrane potential (Figure 3A, Figure S3E), while NAMPT downregulation increased endothelial membrane potential (Figure 3B, Figure S3F). The permeation of FITC-dextran across endothelial cells significantly increased with NAMPT overexpression and decreased with NAMPT knockdown (Figure 3C-D). Moreover, NAMPT upregulation disrupted VE-cadherin distribution at cell junctions and reduced its expression, whereas VE-cadherin levels increased and became more continuous with NAMPT knockdown (Figure 3E, Figure 3H-I). In vitro TEM experiments demonstrated that Dil-labeled THP-1 cells increased with NAMPT (Figure 3F-G), confirming that increased vascular permeability promotes leukocyte TEM. These findings illustrated that NAMPT, which is highly expressed in endothelial cells during periodontitis, enhances endothelial permeability and promotes leukocyte TEM in vitro.

### NAMPT promotes endothelial cell lipid synthesis via FASN

**Figure 4:**
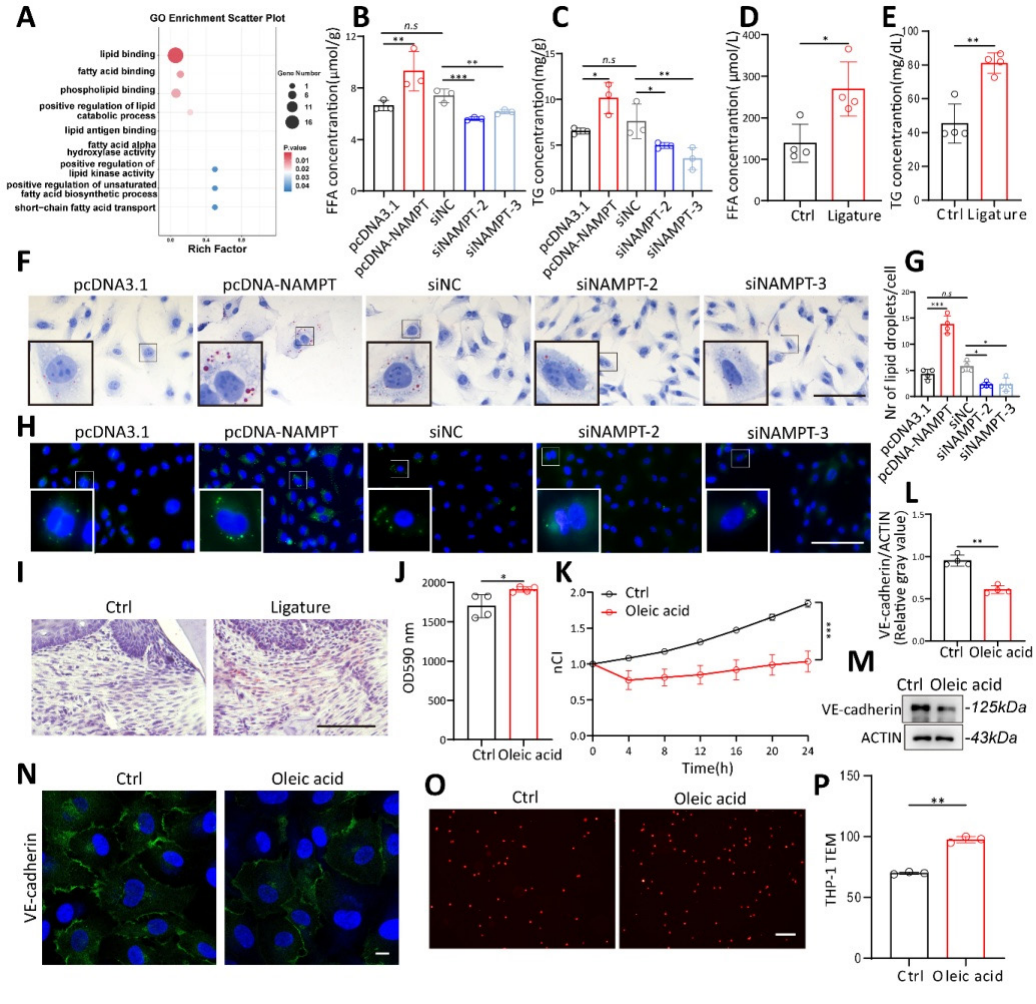
** Elevated lipogenesis promotes HUVEC permeability.** (A) Gene Ontology (GO) analysis of NAMPT overexpressing HUVEC showed that NAMPT affects multiple lipid metabolism related pathways. (B-C) Intracellular triglycerides (TG) and free fatty acids (FFA) concentration after NAMPT overexpression and knockdown, n=3. (D-E) Serum triglycerides (TG) and free fatty acids (FFA) concentration of periodontitis and control mice, n=4. (F) Microscopy observation of Oil Red O staining in NAMPT overexpression and knockdown HUVEC. Scale bars: 100µm. (G-H) Fluorescence microscopy observation and statistical analysis of BODIPY staining in NAMPT overexpression and knockdown HUVEC, n=4. Scale bars: 100µm. (I) Oil Red O staining of periodontal tissue in periodontitis and control mice. Scale bars: 100µm. (J) FITC-dextran density permeated across HUVEC monolayers after exposure to Oleic acid, n=4. (K) The effect of Oleic acid on the impedance of HUVECs in the RTCA system at different time points, n=3. (L-M) Western blot analysis and quantification of VE-cadherin in Oleic acid treated HUVECs, n=4. (N) Immunofluorescence analysis of VE-cadherin in Oleic acid treated HUVECs. Scale bars: 10µm. (O-P) The number of Dil-labeled THP-1 cells passing through oleic acid stimulated endothelial cells was observed under a fluorescence microscope and statistically analyzed, n=3. Error bars indicate SEM. Two-tailed unpaired Student's *t* test was performed between two groups. For multiple comparisons, one-way ANOVA followed by Turkey's test was used. n.s, not significant, *P < 0.05, **P < 0.01, ***P < 0.001.

To further explore the mechanisms by which NAMPT regulates HUVEC permeability, transcriptome sequencing was performed on three pairs of NAMPT-overexpressing endothelial cells. Gene Ontology (GO) analysis showed that NAMPT affects multiple lipid metabolism pathways, including positive regulation of lipid binding, fatty acid binding, unsaturated fatty acids biosynthetic process, and short fatty acids transport (Figure 4A). To validate the relevance of NAMPT to lipid metabolism, intracellular triglycerides (TG) and free fatty acids (FFA) were measured. TG and FFA increased with overexpression of NAMPT (Figure 4B-C), and the opposite was observed when NAMPT was downregulated. Interestingly, TG and FFA serum levels were also increased in periodontitis mice (Figure 4D-E). BODIPY and Oil Red O staining were used to visualize intracellular lipid droplets directly. NAMPT led to intracellular accumulation of lipid droplets obviously (Figure 4F-H). Oil red O staining of mouse periodontal gingival also showed an increase of lipid droplets in periodontitis group (Figure 4I). This suggests that NAMPT promotes cellular lipid metabolism, especially lipogenesis. To further confirm the role of lipogenesis in endothelial permeability, oleic acid, a kind of free fatty acid, was added to HUVECs. Oleic acid treatment increased the intracellular lipid content (Figure S4A-D), enhanced FITC-Dextran permeation (Figure 4J), reduced HUVEC membrane potential (Figure 4K, Figure S4E), decreased VE-cadherin expression (Figure 4L-N), and facilitated leukocyte TEM in vitro (Figure 4O-P).

**Figure 5:**
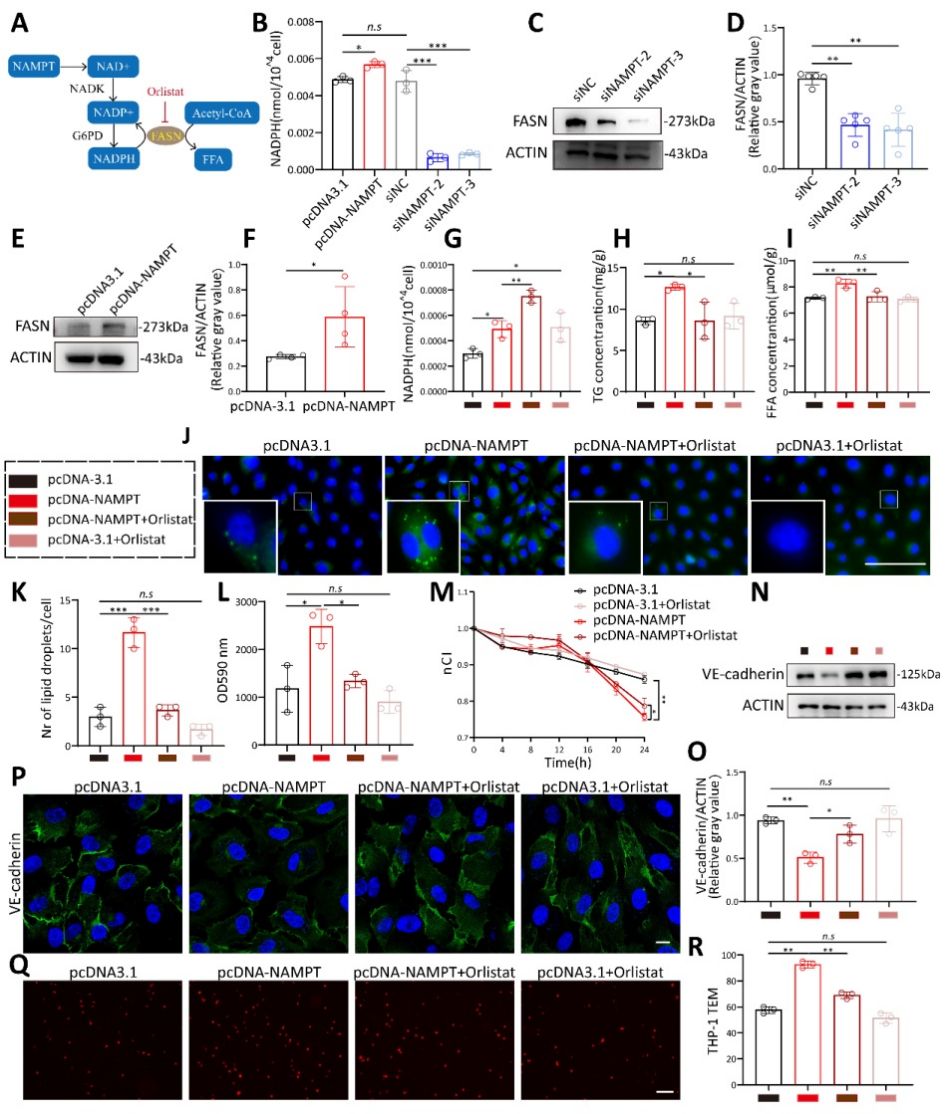
** NAMPT promotes HUVEC lipogenesis and permeability via FASN.** (A) Diagram illustrating the involvement of NAMPT in lipogenesis. (B) NADPH concentrations following NAMPT overexpression and knockdown, n=3. (C-D) Western blot analysis and quantification of FASN protein levels following NAMPT knockdown, n=5. (E-F) Western blot analysis and quantification of FASN protein levels following NAMPT overexpression, n=4. (G) NADPH concentration following NAMPT overexpression with or without orlistat treatment, n=3. (H-I) Intracellular triglycerides (TG) and free fatty acids (FFA) concentrations following NAMPT overexpression with or without orlistat treatment, n=3. (J-K) Fluorescence microscopy observation and statistical analysis of BODIPY staining following NAMPT overexpression with or without orlistat treatment, n=3. Scale bars: 100µm. (L) FITC-dextran density permeated across HUVEC monolayers following NAMPT overexpression with or without orlistat treatment, n=3. (M) Effect of NAMPT overexpression with or without orlistat treatment on the impedance of HUVECs in the RTCA system at different time points, n=4. (N-O) Western blot analysis of VE-cadherin in HUVECs following NAMPT overexpression with or without orlistat treatment, n=3. (P) Immunofluorescence of VE-cadherin in HUVECs overexpressing NAMPT together with orlistat treatment. Scale bars: 10µm. (Q-R) Representative images and quantitative analysis of Dil-labeled THP-1 cells passing through endothelial cells following NAMPT overexpression with or without orlistat treatment, n=3. Error bars indicate SEM. Two-tailed unpaired Student's t test was performed between two groups. For multiple comparisons, one-way ANOVA followed by Turkey's test was used. n.s, not significant, *P < 0.05, **P < 0.01, ***P < 0.001.

As a rate-limiting enzyme in NAD biosynthesis, NAMPT boosts intracellular NAD synthesis which can be transformed into NADP under the catalysis of NADK and then into NADPH (Figure 5A). The NAD+/NADH ratio, NADP+, and NADPH concentrations changed synchronously with NAMPT (Figure 5B, Figure S4G-H). Fatty acid synthase (FASN), a key enzyme in de novo lipogenesis, catalyzes the synthesis of free fatty acids (FFAs), such as palmitic acid and oleic acid, from acetyl-CoA, utilizing NADPH as a reducing agent. It has been demonstrated that NAD kinase (NADK) phosphorylates NAD to synthesize NADP, thereby promoting lipid synthesis via FASN^25^. Given its role as a key regulator of NADP production, we hypothesized that NAMPT may influence FASN levels to regulate lipogenesis, thereby promoting endothelial cell permeability. Western blot showed that FASN decreased when NAMPT was knocked down (Figure 5C-D) and increased when NAMPT was overexpressed (Figure 5E-F). FASN inhibitor orlistat treatment increased cellular NADPH concentration and partially rescued the depletion of NADPH by FASN (Figure 5G). Orlistat inhibited lipid droplet synthesis (Figure 5J-K), and the concentrations of FFA and TG induced by NAMPT overexpression (Figure 5H-I). These suggested that NAMPT may promote intracellular lipid synthesis via FASN. Finally, we treated NAMPT-overexpressing endothelial cells with orlistat and performed permeability-related experiments. Orlistat treatment successfully rescued the increased endothelial cell permeability caused by NAMPT overexpression (Figure 5L-M, Figure S4F), enhanced VE-cadherin expression (Figure 5N-P) and decreased leukocyte transmigration across HUVEC (Figure 5Q-R). These results indicated that the increased endothelial cell permeability due to NAMPT overexpression is associated with its promotion of FASN-mediated lipid synthesis, and the FASN inhibitor orlistat inhibits NAMPT-induced endothelial permeability in vitro.

### FASN increases endothelial cell permeability by promoting VE-cadherin phosphorylation through the ERK pathway

**Figure 6:**
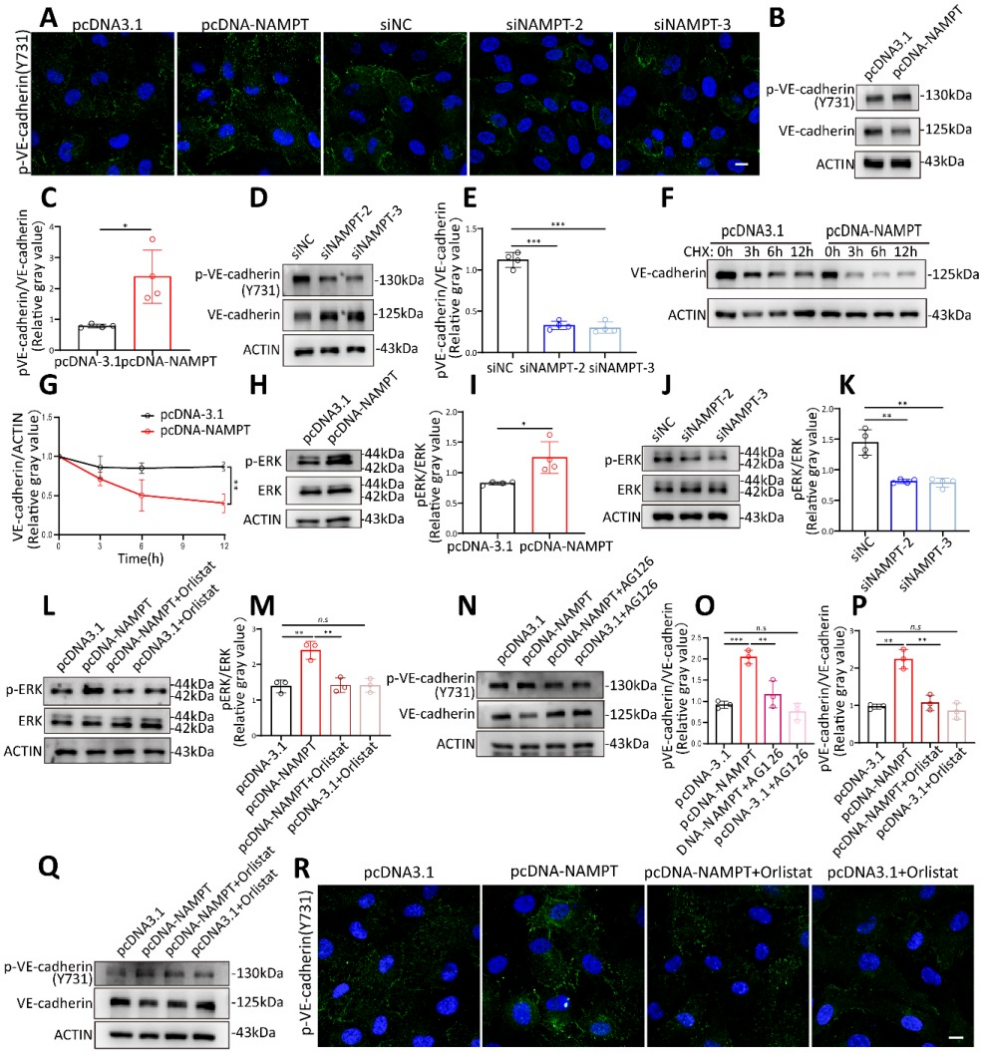
** FASN promoted VE-cadherin phosphorylation through ERK Pathway.** (A) Immunofluorescence analysis demonstrating increased Phospho-VE-cadherin (Tyr731) levels following NAMPT overexpression and decreased levels after NAMPT knockdown. Scale bars: 10µm. (B-E) Western blot analysis and quantification of Phospho-VE-cadherin (Tyr731) levels following NAMPT overexpression or knockdown, n=4. (F-G) Western blot analysis and quantification of VE-cadherin after NAMPT overexpression in HUVEC with cycloheximide (CHX,100 µg/ml) treatment at different time points, n=3. (H-K) Western blot analysis and quantification of phosphorylated ERK (p-ERK) levels following modulation of NAMPT expression, n=4. (L-M) Western blot analysis and quantification of p-ERK levels following NAMPT overexpression, with or without orlistat treatment, n=3. (N-O) Western blot analysis and quantification of Phospho-VE-cadherin (Tyr731) levels following NAMPT overexpression, with or without AG126 treatment, n=3. (P-Q) Western blot analysis and quantification of Phospho-VE-cadherin (Tyr731) levels following NAMPT overexpression, with or without orlistat treatment, n=3. (R) Immunofluorescence analysis demonstrating that orlistat reverses the elevation of Phospho-VE-cadherin protein level caused by NAMPT overexpression. Scale bars: 10µm. Error bars indicate SEM. Two-tailed unpaired Student's *t* test was performed between two groups. For multiple comparisons, one-way ANOVA followed by Turkey's test was used. n.s, not significant, *P < 0.05, **P < 0.01, ***P < 0.001.

It has been reported that phosphorylation of VE-cadherin is a key determinant of endothelial junction integrity, leading to vascular leakage^26^, ^27^. VE-cadherin phosphorylation was detected by western blot and immunofluorescence. Results showed that both oleic acid treatment and NAMPT overexpression promote VE-cadherin phosphorylation at Y731 (Figure 6A-E, Figure S5B-D). Phosphorylation status could affect protein stability and decrease its expression^26^, ^28^. VE-cadherin stability was further evaluated by applying cycloheximide treatment at different time points after NAMPT overexpression (Figure 6F-G). NAMPT overexpression led to an accelerated degradation rate of VE-cadherin, indicating that NAMPT decreases VE-cadherin stability by promoting its phosphorylation. KEGG enrichment analysis revealed that NAMPT overexpression leads to alterations in multiple cellular pathways (Figure S5A), among which the MAPK signaling pathway is involved. Western blot analysis revealed that oleic acid treatment and NAMPT overexpression resulted in the activation of ERK, a key component of the classic MAPK pathway, and an increase in its phosphorylation (Figure 6H-I, Figure S5E-F). While knockdown of NAMPT inhibited ERK phosphorylation in HUVECs (Figure 6J-K). Orlistat effectively rescued ERK activation induced by NAMPT (Figure 6L-M). Furthermore, we validated the role of the FASN-ERK axis in regulating VE-cadherin phosphorylation. Both the ERK inhibitor AG126 and orlistat treatment significantly inhibited VE-cadherin phosphorylation resulting from NAMPT overexpression (Figure 6N-Q). Additionally, AG126 also suppressed VE-cadherin phosphorylation induced by oleic acid treatment (Figure S5G-H). These results demonstrated that NAMPT activates the ERK pathway by increasing intracellular FASN-mediated lipid synthesis, which increases VE-cadherin phosphorylation, decreases its stability, and ultimately leads to increased endothelial cell permeability.

### Orlistat inhibited periodontal vascular leakage and bone resorption in vivo

**Figure 7:**
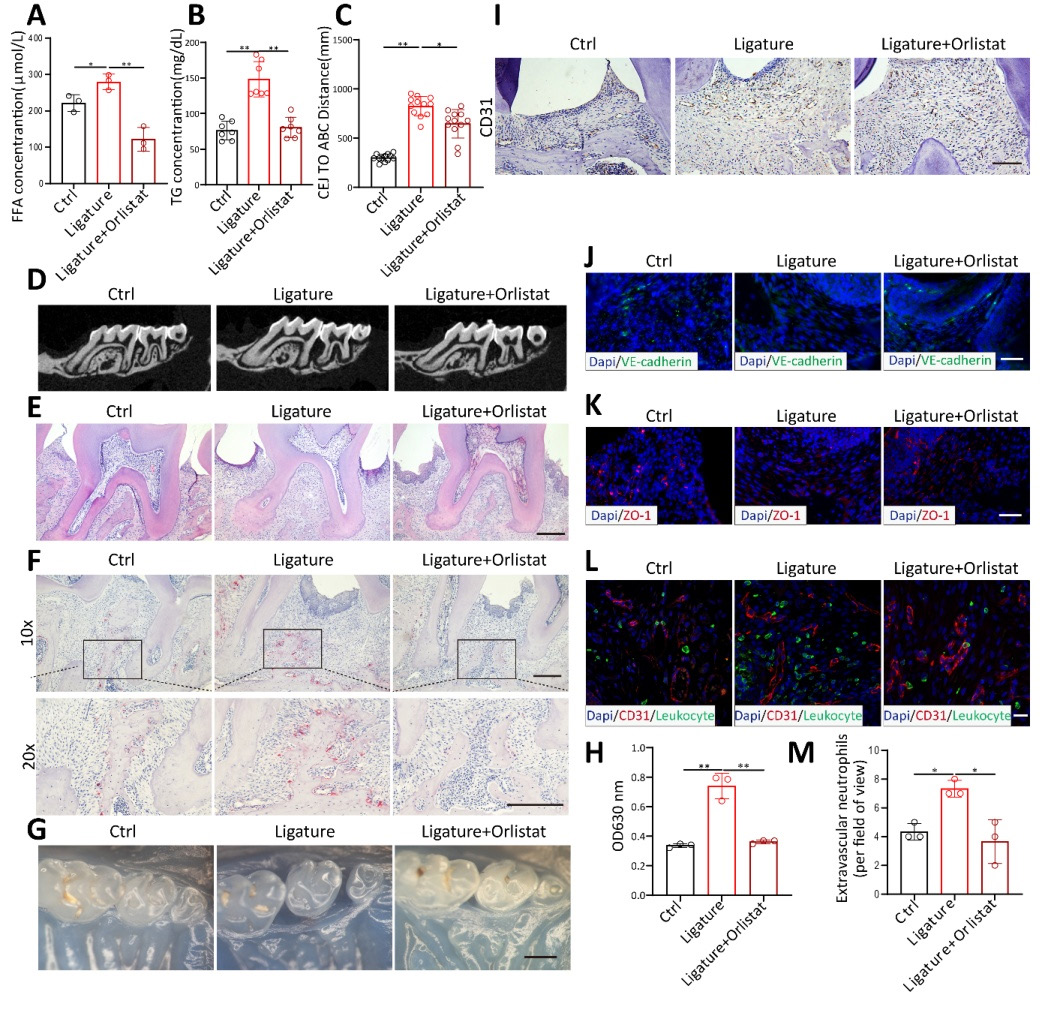
** Orlistat inhibited periodontal vascular leakage and bone resorption in vivo.** (A-B) Measurement of serum TG (n=3) and FFA (n=7) concentrations in periodontitis mice after orlistat treatment. (C-D) Micro-CT analysis of alveolar bone showing the distance from the cemento-enamel junction (CEJ) to the alveolar bone crest (ABC) in orlistat-treated periodontitis mice, n=12. (E-F) hematoxylin and eosin(H&E)-stained and tartrate-resistant acid phosphatase (TRAP)-stained paraffin sections in orlistat-treated periodontitis mice. Scale bars: 200µm. (G-H) Residual Evans Blue dye in periodontal tissue of orlistat-treated periodontitis mice and its quantitative analysis, n=3. Scale bars: 500µm. (I) Immunohistochemistry for CD31 in periodontitis mice treated with Orlistat. Scale bars: 100µm. (J-K) Immunofluorescence of the junction marker VE-cadherin(green) and ZO-1(red) after orlistat treatment in periodontitis mice. Scale bars: 100µm. (L-M) Laser confocal microscope observation and quantitative detection of S100A9(green) labeled neutrophils transmitted through CD31(red) labeled vessels after orlistat treatment in periodontitis mice, n=3. Scale bars: 20µm. Two-tailed unpaired Student's *t* test was performed between two groups. For multiple comparisons, one-way ANOVA followed by Turkey's test was used. Error bars indicate SEM. *P < 0.05, **P < 0.01.

To verify whether orlistat inhibits periodontal vascular permeability in vivo, orlistat was administered by gavage every other day during the induction of periodontitis in mice. Orlistat significantly diminished FFA (Figure 7A) and TG (Figure 7B) levels in mouse blood. CT and HE staining showed that orlistat decreased the distance from the cemento-enamel junction (CEJ) to the alveolar bone crest (ABC) (Figure 7C-E). The number of TRAP-positive osteoclasts was markedly reduced by orlistat (Figure 7F). This proved that orlistat inhibited alveolar bone resorption in mice with periodontitis. Periodontal Evans Blue permeation decreased in periodontal tissue of PD mice after Orlistat treatment (Figure 7G-H). CD31 staining showed altered vascular morphology of periodontal tissues, with a reduced number of dilated blood vessels in orlistat-treated mice (Figure 7I). Immunofluorescence staining demonstrated that VE-cadherin and ZO-1 expression was enhanced after orlistat treatment (Figure 7J-K). Conversely, leukocyte infiltration in the periodontal tissue decreased following orlistat treatment (Figure 7L-M). These results indicated that orlistat strengthens the vascular barrier integrity of periodontal tissues, reduces leukocyte infiltration, and ultimately inhibits periodontitis-induced bone resorption.

## Discussion

Vascular abnormality of periodontal tissue is one of the important pathological changes of periodontitis^29^. Abnormal vascularization and vascular permeability have been observed in local periodontal tissue in periodontitis previously^30^, ^31^. Despite recent studies that have explored that Porphyromonas gingivalis and LPS induced angiogenesis and IL-6 secretion^32^, the specific mechanisms and the impact of these vascular changes on the progression of periodontal disease remain largely unknown. To explore the key molecules that regulate vascular leakage in periodontitis, we conducted GEO database analysis and intersected with the genes changed in the endothelial subcluster in scRNA-seq. COL15A1 and COL4A2, both types of collagen, are strongly associated with anti-angiogenesis^33^. C-terminal fragment of COL4A2 called canstatin inhibits proliferation, migration and tube formation of HUVEC^34^, ^35^. While previous studies have linked COL15A1 to alveolar vascular remodeling^36^, evidence supporting its role in promoting endothelial cell permeability remains limited. However, prior studies have shown that high circulating levels of NAMPT have been reported as a maker of endothelial dysfunction, vascular inflammation, and atherosclerosis^37^, ^38^. Hence, we wonder if NAMPT could contribute to vascular abnormality of periodontitis. In this study, we provide evidence suggesting that periodontitis raised vascular permeability, which results in increased leukocyte TEM and infiltration in periodontal tissue, and NAMPT is increased in endothelial cells of periodontitis tissue. NAMPT upregulates endothelial cell permeability by increasing cellular lipid synthesis via FASN, which can promote by phosphorylating VE-cadherin via the ERK pathway. Using FASN inhibitor orlistat, we further demonstrate its effect on suppression of vascular leakage and bone resorption of periodontitis in vivo. Collectively, these observations suggest that interfering NAMPT-mediated lipid synthesis might be a novel therapeutic target for periodontitis.

NAMPT, an enzyme involved in cellular metabolism, exhibits a complex dual-edged effect, simultaneously acting as an inhibitor of aging and a promoter of inflammation^39^. Prior reports demonstrating that pharmacological inhibition of NAMPT by FK886 reduced inflammation and keratinocyte death in chronic skin inflammation models^40^. FK866 could also suppress inflammation-associated tumorigenesis and ameliorate DSS-induced colitis and in mice^13^. Consistent with this view, we hypothesize that NAMPT may play a role in increased vascular permeability in periodontitis. Our study showed that NAMPT was highly expressed in the endothelial cells in periodontitis gingival compared with the healthy. TNFα treatment increased NAMPT levels in HUVEC. This indicated that NAMPT may act as a key regulator in periodontal vascular permeability. Additionally, NAMPT overexpression promotes HUVEC permeability, characterized by increased FITC-dextran permeation, decreased impedance, and reduced VE-cadherin expression. The number of leukocytes migrating across endothelial cells also increased with NAMPT overexpression. These results revealed that NAMPT is highly expressed in periodontitis, which promotes endothelial permeability and increased leukocyte TEM. However, the underlying mechanism linking NAMPT to endothelial permeability is not known.

Research suggests that NAMPT is involved in regulating lipid metabolism^41^. NAMPT transgene mice showed an aggravated atherosclerotic inflammation in the prior report^42^. Additionally, de novo lipogenesis required the involvement of NAMPT in prostate cancer cells^43^. Here we provide evidence that increased NAMPT affects multiple lipid synthesis-related pathways in cells, indicated by GO enrichment analysis of transcriptome sequencing and NAMPT overexpression increases endothelial cell lipid droplet content. These findings suggest that abnormal lipid metabolism may play a role in NAMPT-induced endothelial cell permeability. Excess lipid accumulation impairs endothelial cell barrier function and contributes to vascular leakage^44^. It has been reported that free fatty acids (FFAs) can promote endothelial cell dysfunction^45^, ^46^. This is verified by our finding that endothelial cell permeability increased after treatment with oleic acid. These results further demonstrated the promotion of vascular permeability by NAMPT is related to the increase in intracellular lipid production, suggesting that inhibition of cellular lipogenesis can mitigate the adverse effects of NAMPT. However, the mechanism of how NAMPT regulates cellular lipid synthesis remains unknown.

NAMPT is a key enzyme involved in the biosynthesis of nicotinamide adenine dinucleotide (NAD+), a crucial coenzyme in cellular redox reactions and a substrate for NAD-dependent enzymes^39^. It has been previously reported that NAD+ can be phosphorylated to NADP+ by NAD kinases (NADKs)^25^. Thus, NAD+ and NADP+ can be reduced to NADH or NADPH via dehydrogenases respectively^47^. Known as a regulator of cellular energy metabolism, the NAD/NADH redox couple regulates glycolysis and mitochondrial oxidative phosphorylation. Subsequent studies have shown that NAD^+^ depletion suppressed glycolysis and dampened ATP synthesis, thus meeting the energetic demands of cells^48^, ^49^. By contrast, NADP(H) is involved in supporting the biosynthesis of fatty acids^50^. A recent study verified that Folate-mediated serine catabolism enhances hepatic cellular NADPH production thereby promoting hepatic lipid synthesis^51^. We observed that NAMPT overexpression simultaneously increased the NAD(H) pool and NADP(H) pool, which is consistent with previous studies^52^, ^53^. Additionally, NADPH serves as a reducing agent of FASN, providing electrons for the reduction reactions during fatty acid synthesis. The expression of FASN also increased with NAMPT in our study. It is suggested to us that NAMPT increases the cellular NADP(H) pool and regulates FASN expression thereby increasing cellular lipid synthesis. To further validate the role of FASN, Orlistat, a well-known inhibitor of FASN^54^, was used to block NAMPT-induced lipogenesis. Orlistat abolished NAMPT-induced lipogenesis and partially inhibited the consumption of NADPH, resulting in increased intracellular NADPH levels. Moreover, orlistat decreased NAMPT induced endothelial cell permeability, and inhibited leukocyte TEM. Our study revealed that NAMPT promotes HUVEC lipogenesis through regulation of NADPH-dependent enzyme FASN, which may be one of the important mechanisms by which NAMPT induces endothelial permeability.

Many studies have shown that the integrity of the endothelium is maintained by intercellular junction proteins, such as VE-cadherin, CLND5, PECAM-1, etc.^55^, ^56^. Of note, it has previously been reported that phosphorylation of VE-cadherin can lead to its degradation and disrupt endothelial barrier integrity^57^. Similarly, our data suggested an increase in VE-cadherin phosphorylation in NAMPT overexpressing HUVEC. Previous studies have reported that activation of the ERK pathway can phosphorylate cytoskeletal proteins and disrupt tight junctions^58^, ^59^. Meanwhile, evidence showed that lipogenesis is highly correlated with ERK pathway activation. On the one hand, FASN overexpression promotes lipid accumulation and ERK pathway activation^60^. Orlistat treatment diminished phosphorylation of ERK pathways in colorectal cancer (CRC) cell lines^61^. On the other hand, ERK can be activated by FFAs which are produced by FASN^62^, ^63^. In this study, the ERK pathway was enriched in KEGG analyses of NAMPT overexpression transcriptome sequencing and its activation was further verified by western blotting. Meanwhile, orlistat treatment inhibited the phosphorylation of ERK caused by NAMPT. Our data indicate that NAMPT induced lipogenesis affects VE-cadherin phosphorylation through ERK pathway. Finally, orlistat treatment was used in vivo. Orlistat decreased endothelial cell permeability, and inhibited leukocyte trans-endothelial migration in periodontitis mice. Interestingly, orlistat also inhibited alveolar bone resorption in mice, demonstrating the pathogenic effect of FASN mediated vascular endothelial cell leakage on bone resorption.

This study has some limitations. Firstly, orlistat administration was able to partially mitigate alveolar bone resorption. This may be attributed to the fact that, while orlistat inhibits periodontal vascular permeability by suppressing excessive immune responses, bacteria and inflammatory factors released by other cells can still contribute to local periodontal bone destruction. This finding suggests that the clinical effectiveness of orlistat could be enhanced when combined with localized elimination of pathogenic bacteria. Despite this, we cannot overlook the significant influence of vascular changes on the progression of periodontitis. Secondly, as a conduit for transporting cells, nutrients, and metabolic waste, the vascular system's role in our study was primarily confined to its regulatory effects on local periodontal tissue. However, given the reported associations between periodontitis and various systemic diseases, further investigation into vascular changes in periodontitis could offer valuable insights into its connection with cardiovascular diseases.

In conclusion, our study highlights the significance of vascular leakage in the pathogenesis of periodontal disease for the first time. NAMPT is a key molecule in the regulation of endothelial cell permeability, which up-regulates endothelial cell lipid synthesis via FASN and therefore phosphorylating VE-cadherin via the ERK pathway. Furthermore, orlistat inhibits the increase of vascular permeability in a periodontitis environment and thus decreases periodontal bone resorption. Our study suggests a potential use of orlistat in the periodontitis, which may offer novel insights into the therapeutic strategies for managing periodontal disease.

## Methods

### Animal experiments and clinical sample collection

To eliminate the influence of estrogen, ligatures were placed around the second maxillary molars of 12 male C57BL/6 mice (8-10 weeks old) to induce periodontal inflammation. A sham-operated group of mice served as controls. After 14 days, half of the mice were euthanized and the maxillae were dissected. Samples were fixed in 4% paraformaldehyde for 24 hours at 4°C. Fixed tissues were subjected to micro-CT scanning to evaluate alveolar bone loss. Following CT imaging, tissues were decalcified in 10% EDTA for 2 weeks. For the remaining mice, Evans blue dye (20 mg/kg, Merck Millipore, Germany) was injected via the tail vein. Mice were euthanized after saline perfusion 30 minutes post-injection. Evans blue dye extravasation was quantified by extracting the dye from the gingival tissues using formamide and measuring the absorbance at 620 nm. Tissues were then fixed and decalcified for histological analysis. Orlistat (10mg/kg, MCE, USA) was administered via oral gavage every other day. All animal procedures were approved by the Institutional Animal Care and Use Committee (IACUC: 2308008) and conducted following relevant guidelines and regulations.

6 healthy and 20 periodontitis gingival tissue samples were collected from patients at the Department of Periodontology, Affiliated Stomatological Hospital of Nanjing Medical University. Healthy samples were obtained from sites with no clinical signs of inflammation, bleeding on probing (BOP), or periodontal pocketing during crown lengthening procedures. Periodontitis samples were collected from sites where inflammation did not completely subside after basic periodontal treatment with periodontal pockets ≥5 mm that required flap surgical surgery. The samples were categorized based on the stages of periodontitis, with stages I and II representing mild periodontitis, and stages III and IV representing severe periodontitis. None of the volunteers had systemic diseases. 4% paraformaldehyde was used for fixation of the samples immediately and then processed for routine histological analysis. The study was conducted with ethical approval (Ethics No. [PJ2023-101-001]).

### Immunohistochemistry and immunofluorescence staining

For immunohistochemistry, tissue sections were first subjected to deparaffinization, followed by gradual rehydration, and then treated with heat-induced antigen retrieval using citrate buffer (pH 6.0). 10% bovine serum albumin (BSA) was used for blocking and sections were incubated with anti-CD31 antibody (1:1000, abcam, UK), anti-PECAM1 antibody (1:500, ABclonal, China), and anti-NAMPT antibody (1:500, Proteintech, China) overnight at 4°C. Detection was carried out using a biotinylated secondary antibody and a DAB substrate. Images were acquired with a Leica DM4000 fluorescence microscope.

For immunofluorescence, 4µm sections were similarly processed for antigen retrieval and blocking. Pre-treated cells were seeded onto 14 mm coverslips and, following fixation, were blocked with 10% BSA. They were incubated with primary antibodies: anti-CD31 (1:500, Proteintech, China), anti-PECAM1 (1:500, ABclonal, China), anti-S100A9 (1:200, Proteintech, China), and anti-VE-cadherin (1:400, Proteintech, China) overnight at 4°C. After washing, sections were incubated with Alexa Fluor-conjugated secondary antibodies (1:500, Proteintech, China) for 1 hour at room temperature. Nuclei were counterstained with DAPI (1:1000, Beyotime, China) for 15 minutes. The slides were mounted with anti-fade mounting medium. Visualization was performed using a Zeiss LSM710 confocal microscope.

### H&E and TRAP staining

For H&E staining, deparaffinized and rehydrated tissue sections were stained with hematoxylin for 2 minutes, followed by differentiation in 1% acid alcohol. The sections were then dehydrated to 80% ethanol and stained with eosin for 2 minutes. Subsequently, they were further dehydrated through graded alcohols, cleared in xylene, and cover-slipped.

For TRAP staining, decalcified tissue sections were incubated with TRAP staining solution at 37°C for 20 minutes according to the manufacturer's protocol to identify osteoclasts. The sections were counterstained with hematoxylin, and images were captured using a Leica DM4000 fluorescence microscope.

### Cell culture and transfection

THP-1 cells were cultured in RPMI-1640 medium supplemented with 10% FBS, 1% P/S. HUVECs were cultured in Endothelial Cell Growth Medium (ECM, Science Cell, USA) supplemented with 5% fetal bovine serum (FBS), 1% P/S and endothelial cell growth supplements. Both cell types were incubated at 37°C in 5% CO2. Experiments were conducted using cells between passages 3 and 6 for HUVECs and at a density of 1×10^^6^ cells/mL for THP-1 to ensure consistent cellular behavior. Orlistat (MCE, USA) was administered at a concentration of 40 μM for 24 hours, TNFα (MCE, USA) at 10 ng/mL for 12 hours, AG126 (MCE, USA) at 10 μM for 24 hours, and oleic acid (MCE, USA) at 100 nM for 24 hours.

For HUVEC transfection, cells were seeded in 6-well plates at a density of 2×10^^5^ cells per well and transfected NAMPT plasmid (Transheep, China) and siRNA (Generay, China) at 70-80% confluence using jetPRIME ® Versatile DNA/siRNA transfection reagent (Polyplus, France) according to the manufacturer's instructions. siRNA sequences were listed in Table S2, and we selected siNampt-2 and siNampt-3 for the following experiments based on the knockout efficiency. Transfection efficiency was confirmed by RT-qPCR and Western blot analysis 48 hours post-transfection.

### Cell culture in the real-time cell electric impedance sensing system (RTCA, xCELLigence)

The RTCA-DP xCELLigence system (Roche Applied Science, USA), which tracks electrical impedance signals, enables real-time monitoring of cell growth on microelectrode-coated plates. Changes in HUVEC monolayer permeability are reflected in the impedance readout, expressed in arbitrary units as the "cell index" (CI). The normalized cell index (nCI) is calculated by dividing the CI at the normalized time point by its baseline value. Transfected HUVECs were seeded into E-plates at a density of 1 × 10^4 cells/well, allowing them to adhere and form a confluent monolayer. After 24 hours, when the HUVECs reached a plateau, cells were stimulated with orlistat, and changes in the cell index (CI) were monitored every 15 minutes for 24 hours. Data analysis was performed using RTCA software version 2.0.

### In vitro endothelial permeability assay

Transfected HUVECs were cultured to confluence on Transwell inserts with a pore size of 0.4 µm (Corning, China). After treatments, FITC-dextran (70 kDa, 2mg/ml, Sigma-Aldrich, USA) was added to the upper chamber and incubated for 1 hour at 37°C. Subsequently, 40 µL of the medium from the lower chamber was collected, and the fluorescence intensity was measured using a Spectra Max microplate reader (SpectraMax i3, Molecular Devices, USA) at 485 nm excitation and 525 nm emission. Permeability was calculated based on the FITC-dextran fluorescence intensity in the lower chamber, reflecting changes in endothelial barrier function.

### Trans-endothelial migration (TEM) assay

HUVECs were grown to confluence on collagen-coated Transwell inserts with a pore size of 8 µm (Corning, China). THP-1 cells were stained using the cell plasma membrane staining kit with DiI (Beyotime, China) for 30 minutes at 37°C. Labeled THP-1 cells (1×10^5 cells/well) were then added to the upper chamber and allowed to migrate for 4 hours at 37°C. After migration, non-migrated cells were removed from the upper chamber, and the cells that had migrated to the lower chamber were observed under Leica inverted microscopy.

### Sequencing analysis

For transcriptome sequencing, three pairs of HUVECs transfected with pcDNA-NAMPT and pcDNA3.1 control were collected. RNA was extracted using Trizol (Vazyme, China) and sequenced on an Illumina NovaSeq 6000 platform (LC-Bio Technology Co., Ltd., China). Differentially expressed genes (DEGs) were identified using the DESeq2 package, with criteria set at fold change (FC) ≥ 2 and P-value ≤ 0.05. Gene Ontology (GO) and Kyoto Encyclopedia of Genes and Genomes (KEGG) pathway enrichment analyses were performed using the clusterProfiler package in R, highlighting significantly enriched biological processes and pathways.

Microarray datasets GSE10334 and GSE16134, RNA sequencing dataset GSE173078, and single-cell RNA sequencing data from GSE164241 were obtained from the GEO database. Differential gene analysis of the microarray datasets was conducted using Geo2R. For the GSE173078 dataset, differential gene expression analysis was performed using the DESeq2 package in R. Single-cell RNA sequencing data were processed using the Cell Ranger pipeline for alignment, filtering, and UMI counting. Downstream analyses, including clustering and differential gene expression, were conducted with the Seurat package in R. To address potential batch effects that could affect the accuracy of single-cell analyses, batch effect correction was applied using the Harmony package. Cell type annotation was carried out using the SingleR package, which compares each cell's gene expression profile to reference datasets to identify the most likely cell type. UMAP plots were generated for data visualization, enabling the identification of cell populations and their gene expression profiles. Single-cell RNA downstream analyses, including clustering and differential gene expression, were conducted with the Seurat package in R. To address potential batch effects that could affect the accuracy of single-cell analyses, batch effect correction was applied using the Harmony package. Cell type annotation was carried out using the SingleR package, which compares each cell's gene expression profile to reference datasets to identify the most likely cell type. UMAP plots were generated for data visualization, enabling the identification of cell populations and their gene expression profiles. TEM activity was calculated using the AUCell package.

### Lipid droplet staining

For Oil Red O staining, cells were first rinsed with PBS, then fixed in 4% paraformaldehyde for 15 minutes at room temperature, and subsequently stained with freshly prepared Oil Red O solution (Beyotime, China) for 10 minutes. After staining, the cells were washed with distilled water and counterstained with hematoxylin for 1 minute to stain the cell nuclei.

For BODIPY staining, HUVECs were incubated with BODIPY 493/503 (1 µg/mL, MCE, USA) for 30 minutes at 37°C, protected from light. Nuclei were counterstained with DAPI. The stained cells were then gently rinsed with PBS, mounted with an anti-fade mounting medium, and visualized using a Leica DM4000 fluorescence microscope to assess lipid droplet accumulation.

### Serum and cellular triglyceride (TG) and free fatty acid (FFA) measurement

Serum and cellular TG levels were measured using triglyceride assay kit (Solarbio, China) and FFA assay kit (Solarbio, China). In brief, blood samples were collected and centrifuged at 3000 rpm for 10 minutes to obtain serum. Cells were lysed in a lysis buffer provided by the kit. Ultrasonic crushing for 1min, centrifuge at 8000rpm for 10min to get the supernatant. Both serum and cell lysates were mixed with the TG assay reagent according to the manufacturer's instructions. The samples were incubated at 65°C for 15 minutes. For FFA measurement, both serum and cell lysates were mixed with the FFA assay reagent according to the manufacturer's instructions. The samples were vortex for 15min, 3000rpm, centrifuge for 10min. Absorbance was measured at 550 nm using a Spectra Max microplate reader (SpectraMax i3, Molecular Devices, USA). FFA and TG concentrations were calculated based on a standard curve.

### Cellular NAD+/NADH and NADP+/NADPH measurement

Cellular NAD+ and NADH levels were measured using a commercial NAD+/NADH assay kit (Beyotime, China). Cells were harvested and lysed in an extraction buffer provided by the kit. The lysates were divided into two portions: one for NAD+ measurement and one for NADH measurement. The NADH portion was heated at 60°C for 30 minutes to decompose NAD+. Both portions were then mixed with the NAD+/NADH assay reagent following the manufacturer's instructions. Samples were incubated at 37°C for 10min. Absorbance was measured at 450 nm using a Spectra Max microplate reader (SpectraMax i3, Molecular Devices, USA). NAD+ and NADH concentrations were calculated based on standard curves prepared with known concentrations of NAD+ and NADH.

Cellular NADP+ and NADPH levels were measured using a commercial NADP+/NADPH assay kit (Solarbio China). Cells were harvested and divided into two portions. Acid extract solution was used for NADP+ extraction and alkaline extract solution was used for NADPH measurement. After ultrasonic 1min (power 200W, ultrasonic 2s, stop for 1s), boil for 5min, supernatant was taken by centrifuge 10000g at 4 ℃ for 10min. The liquid is centrifuged again after neutralization and the supernatant is taken for testing following the manufacturer's instructions. Absorbance was measured at 570 nm using a Spectra Max microplate reader (SpectraMax i3, Molecular Devices, USA). NADP+ and NADPH concentrations were calculated based on standard curves prepared with known concentrations of NADP+ and NADPH.

### Western blotting (WB)

Cells were lysed in RIPA buffer supplemented with protease and phosphatase inhibitors. Protein concentrations were measured using BCA protein assay kit (Beyotime, China). Equal amounts of protein (20-30 µg) were separated by SDS-PAGE and transferred onto PVDF membranes. For 1 hour at room temperature, the membranes were blocked with 5% non-fat milk in TBST (Tris-buffered saline with 0.1% Tween-20). Membranes were then incubated overnight at 4°C with primary antibodies against NAMPT (1:2000, Proteintech, China), VE-cadherin (1: 1000, Proteintech, China), p-VE-cadherin (1: 1000, Affinity, USA), β-actin (1:4000, Affinity, USA), FASN (1:1000, ABclonal, China), ERK (1:1000, Cell Signaling Technology, USA) and p-ERK (1:1000, Cell Signaling Technology, USA). Following washing, HRP-conjugated secondary antibodies were used to incubate the membranes for 1 hour at room temperature. Visualization of bands was achieved with an enhanced chemiluminescence (ECL) detection system, and imaging was performed using the Tanon-5200 Multi Chemiluminescent System. ImageJ software was used to analyze the gray values of protein bands. Relative protein expression levels were calculated by normalizing the gray values to β-actin, which served as an internal control to account for potential variations in sample loading. The normalized data from biological replicates were averaged, and statistical comparisons were made between experimental and control groups. To ensure the reliability and reproducibility of our findings, a minimum of three independent biological replicates were performed for each Western blot experiment.

### Reverse transcriptional quantitative polymerase chain reaction (RT-qPCR)

Using TRIzol reagent (Vazyme, China), total RNA was extracted from cells according to the manufacturer's instructions. This RNA was then reverse transcribed into cDNA with the HiScript III 1st Strand cDNA Synthesis Kit (Vazyme, China). RT-qPCR was carried out on a Roche LightCycler 96 with ChamQ Universal SYBR qPCR Master Mix (Vazyme, China). Primers specific for NAMPT and β-actin were designed and validated for efficiency. The PCR cycling conditions included 5 minutes at 95°C, followed by 40 cycles of 15 seconds at 95°C and 1 minute at 60°C. Relative gene expression levels were calculated using the 2^(-ΔΔCt) method, with normalization to β-actin. To ensure accuracy and reproducibility, all reactions were performed in triplicate. Primers are shown in Table S1.

### Statistical analysis

Statistical analysis was conducted using GraphPad Prism 8 software (GraphPad Software Inc., San Diego, CA, USA). Comparisons between two groups were analyzed using a two-tailed unpaired Student's t-test, with statistical significance defined as a p-value less than 0.05. For multiple comparisons, one-way analysis of variance (ANOVA) followed by Tukey's test was used. Each experiment was independently repeated at least three times to ensure reproducibility and reliability.

### Study approval

All animal experiments followed the guidelines set forth by the NIH Guide for the Care and Use of Laboratory Animals (National Academies Press, 2011). All animal procedures were performed according to the approved protocol by the IACUC of Nanjing Medical University (no. 2308008). According to the guidelines of the Ethics Committee of the Affiliated Stomatology Hospital of Nanjing Medical University, gingival tissue excised during surgery was collected from participants after obtaining their informed written consent.

## Supplementary Material

Supplementary figures and tables.

## Abbreviations

HUVEC: Human Umbilical Vein Endothelial Cells
NAMPT: Nicotinamide phosphoribosyltransferase
TEM: Trans-Endothelial Migration
FASN: Fatty Acid Synthase
NAD+: Nicotinamide adenine dinucleotide
NADH: reduced nicotinamide adenine dinucleotide
NADP+: Nicotinamide adenine dinucleotide phosphate
NADPH: reduced nicotinamide adenine dinucleotide phosphate
TG: Triglycerides
FFA: Free Fatty Acids
