# Supplementary material for: NAMPT Impairs Vascular Permeability in Periodontitis by Influencing FASN-mediated Lipogenesis
Source: Int J Biol Sci. 2025 Mar 31;21(6):2707–24. doi: 10.7150/ijbs.104485 (PMC12035901; doi:10.7150/ijbs.104485)
Supplement: Supplementary file 1 — Supplementary figures and tables. [file ijbsv21p2707s1.pdf]

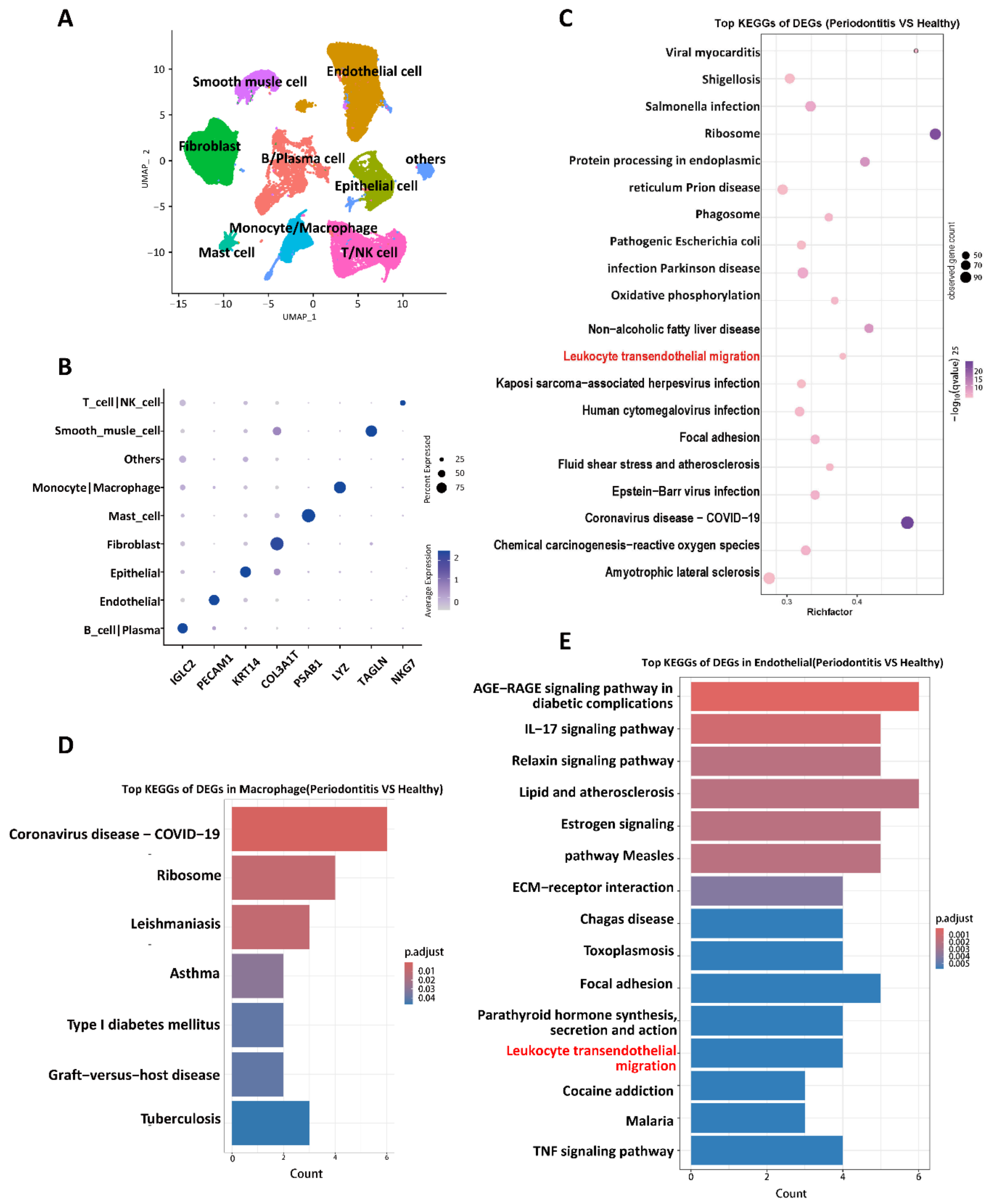

Figure S1. Bioinformatics analysis of single-cell RNA sequencing data from the GSE164241 dataset. (A) UMAP plot displaying sub-clustering of GSE164241. (B) Dot plot illustrating the marker genes for each sub-cluster identified in the GSE164241 dataset. (C) Bubble plot illustrating the top Kyoto Encyclopedia of Genes and Genomes (KEGG) pathways

comparing periodontitis with healthy samples across the entire cluster. (D) Bar plot illustrating the top KEGG pathways comparing periodontitis with healthy samples across the monocyte/macrophage cluster. (E) Bar plot illustrating the top KEGG pathways comparing periodontitis with healthy samples across the endothelial cluster.

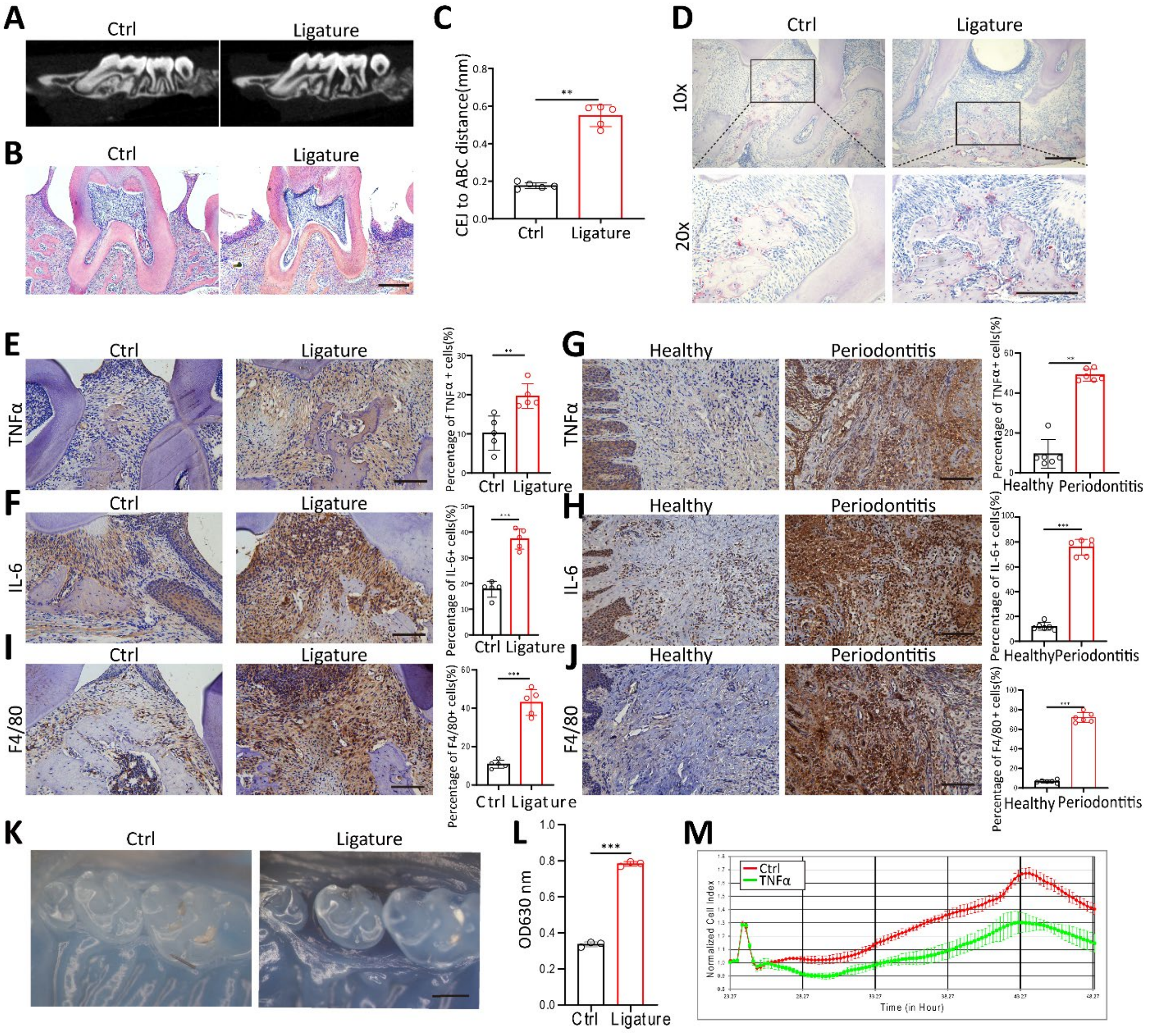

Figure S2. Histological staining of periodontal tissues from human and mouse samples. (A-C) Micro-CT analysis and hematoxylin and eosin (H&E) staining of alveolar bone, demonstrating the distance from the cemento-enamel junction (CEJ) to the alveolar bone crest (ABC) in healthy and periodontitis mice (n = 5). (D) Tartrate-resistant acid phosphatase (TRAP)-stained paraffin sections of healthy and periodontitis mice. Scale bars: 200μm. (E-F) Immunohistochemistry staining and quantifications of inflammatory factor TNFα and IL-6 in healthy and periodontitis mice, n=5. Scale bars: 100μm. (G-H) Immunohistochemistry staining and quantification of inflammatory factors TNF-α and IL-6 in periodontal tissues from healthy

individuals and periodontitis patients, n=6. Scale bars: 100µm. (I-J) Immunohistochemistry staining was performed to evaluate F4/80 expression in periodontal tissues, with quantitative analysis comparing healthy and periodontitis conditions in both mouse (n = 5) and human (n = 6). Scale bars: 100µm. (K) The mouse tail vein was injected with Evans Blue (EB) before sacrificed. The mouse maxilla was collected and observed under a microscope. Scale bars: 500µm. (L) Quantitative detection of EB in the maxillary gingiva of mice, n=3. (M) The impedance of HUVECs in the RTCA system after TNFα treatment at different time points shown in RTCA software2.0, n = 3. Error bars indicate SEM. Two-tailed unpaired Student's *t* test was performed. **P < 0.01, ***P < 0.001.

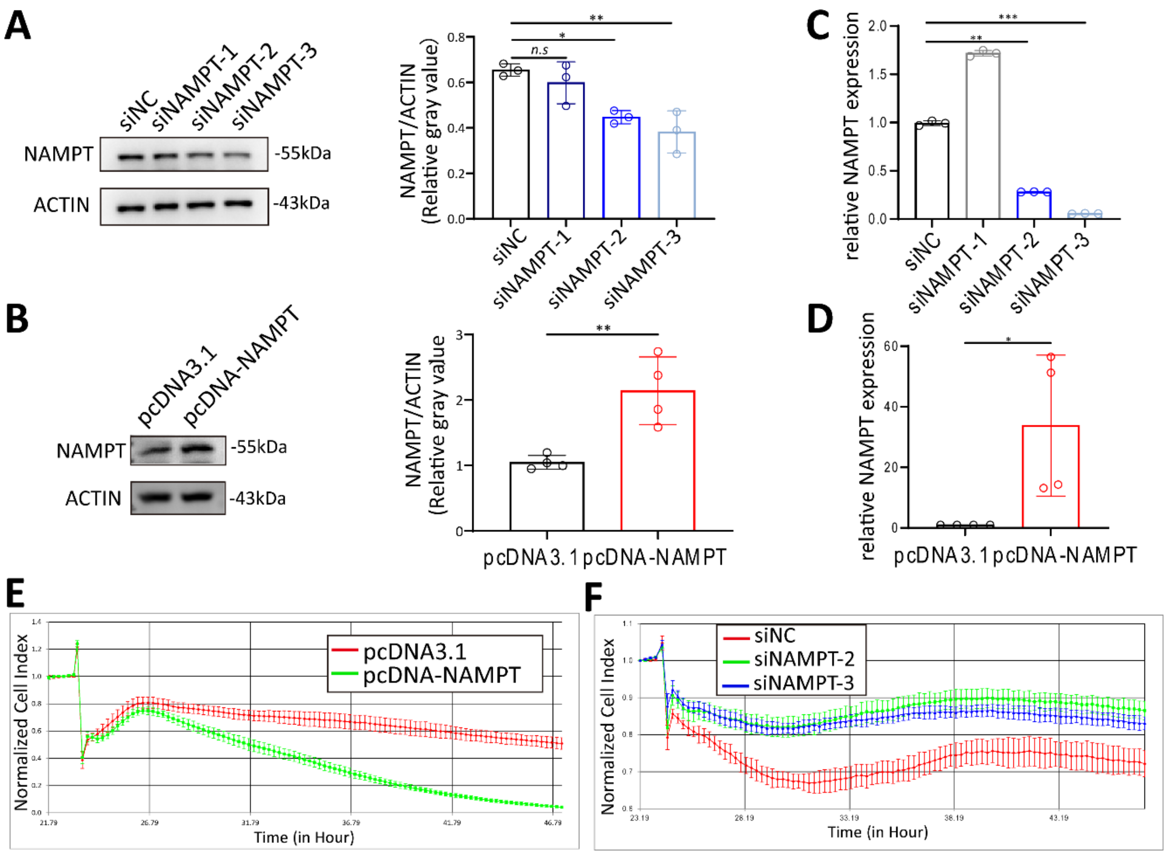

Figure S3. Transfection efficiency of NAMPT in HUVECs. (A-B) Western blot analysis and quantification of NAMPT after NAMPT knockdown (n = 3) and overexpression (n = 4). (C-D) RT-qPCR analysis of NAMPT after NAMPT knockdown (n = 3) and overexpression (n = 4). (E)The effect of NAMPT overexpression on the impedance of HUVECs in the RTCA system at different time points in RTCA software2.0 (n = 3). (F) The effect of NAMPT knockdown on the impedance of HUVECs in the RTCA system at different time points in RTCA software2.0 (n = 3). Error bars indicate SEM. For comparisons between two groups, two-tailed unpaired Student's *t* test was performed. For multiple comparisons, one-way ANOVA followed by Turkey's test was used. *P < 0.05, **P < 0.01, ***P < 0.001.

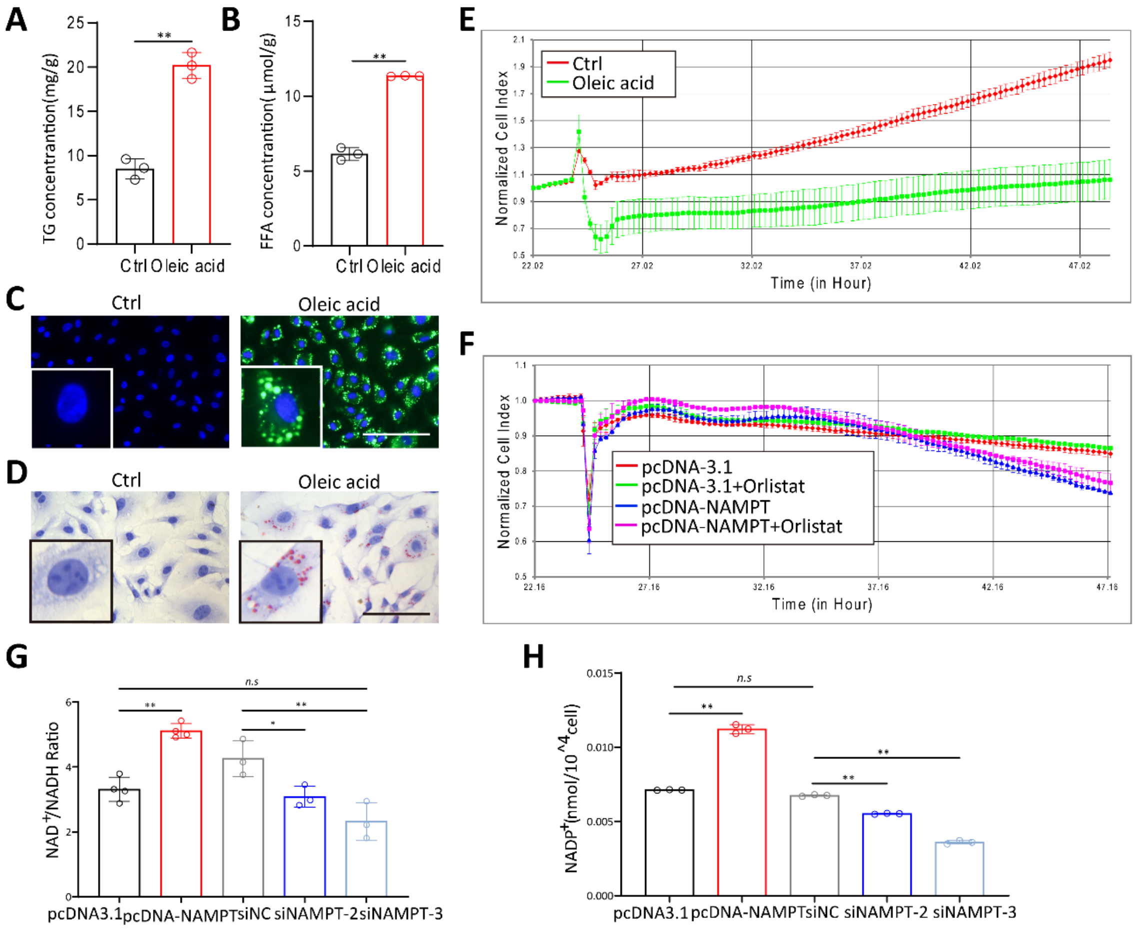

Figure S4. Oleic acid stimulation increased HUVEC lipogenesis. (A-B) Intracellular triglycerides (TG) and free fatty acids (FFA) concentration after oleic acid stimulation, n = 3. (C-D) Oil Red O and BODIPY staining of oleic acid stimulated HUVEC. Scale bars: 100μm. (E) The effect of Oleic acid on the impedance of HUVECs in the RTCA system at different time points in RTCA software2.0, n = 3. (F) Effect of NAMPT overexpression with orlistat treatment on the impedance of HUVECs in the RTCA system at different time points in RTCA software2.0, n = 4. (G) Changes in the NAD+/NADH ratio following NAMPT overexpression and knockdown, n=3. (H)NADP+ concentrations after altering NAMPT expression, n=3.Error bars indicate SEM. For comparisons between two groups, two-tailed unpaired Student’s *t* test was performed. For multiple comparisons, one-way ANOVA followed by Turkey's test was

used. n.s, not significant, *P < 0.05, **P < 0.01.

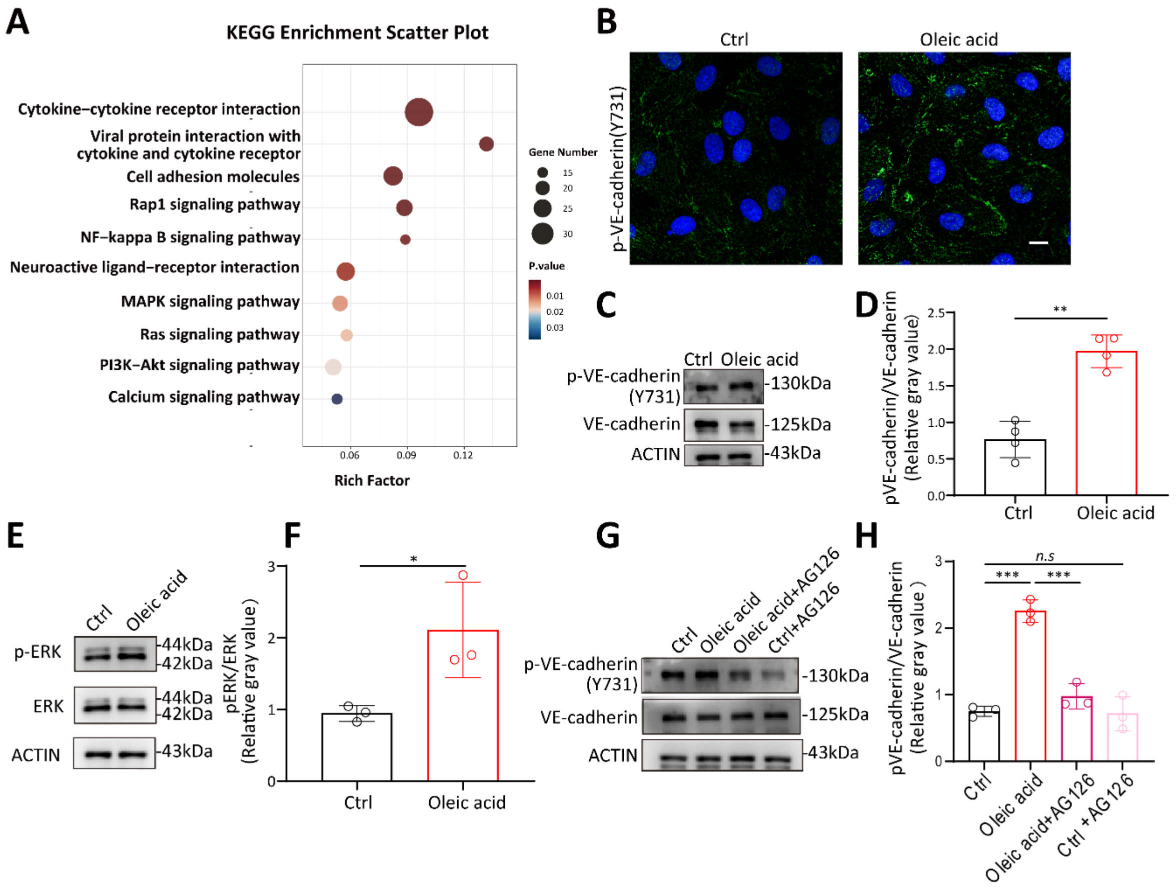

Figure S5. Oleic acid activated ERK Pathway to promoted VE-cadherin phosphorylation. (A) KEGG pathway enrichment analysis indicating that NAMPT impacts multiple pathways. (B) Immunofluorescence showing an increase in Phospho-VE-cadherin (Tyr731) after oleic acid treatment. (C-D) Western blot analysis and quantification demonstrating an increase in Phospho-VE-cadherin (Tyr731) with oleic acid treatment, n=4. (E-F) Western blot analysis showing the expression of ERK and phosphorylated ERK (p-ERK) after oleic acid treatment, n=3. (G-H) Western blot analysis and quantification of Phospho-VE-cadherin (Tyr731) levels following oleic acid stimulation, with or without AG126 treatment, n=3. For comparisons between two groups, two-tailed unpaired Student's *t* test was performed. For multiple comparisons, one-way ANOVA followed by Turkey's test was used. n.s, not significant, *P < 0.05, **P < 0.01, ***P < 0.001.

Table S1：Primers used in this study

Gene	Forward primer	Reverse primer
NAMPT	*CAGCAGCAGAACACAGTACCA*	*ATCGCTGACCACAGATACAGG*
ACTIN	*TCATGAAGTGTGACGTGGACAT*	*CTCAGGAGGAGCAATGATCTTG*

Table S2：siRNA sequence used in this study

Name	Sequence (5’-3’)
siNAMPT-1	*CCUGCGGCAGAAGCCGAGUUCAACA*
siNAMPT-2	*CCACCGACUCCUACAAGGUUACUCA*
siNAMPT-3	*GAUCUUCUCCAUACUGUCUUCAAGA*
